# Human-centered designed communication tools for obesity prevention in early life

**DOI:** 10.1016/j.pmedr.2023.102333

**Published:** 2023-07-22

**Authors:** Erika R. Cheng, Courtney Moore, Lisa Parks, Elsie M. Taveras, Sarah E. Wiehe, Aaron E. Carroll

**Affiliations:** a Indiana University School of Medicine, Department of Pediatrics, Division of Children’s Health Services Research, 410 W. 10^th^ St., Suite 2000, Indianapolis, IN 46220, United States; b Indiana Clinical & Translational Sciences Institute (CTSI), Research Jam, 410 W. 10^th^ St., Suite 1000, Indianapolis, IN 46220, United States; c Kraft Center for Community Health, Massachusetts General Hospital, 125 Nashua St., Boston, MA 02114, United States; d Department of Nutrition, Harvard T.H. Chan School of Public Health, 665 Huntington Ave., Boston, MA 02115, United States; e Indiana University School of Medicine, Department of Pediatrics, Center for Pediatric and Adolescent Comparative and Effectiveness Research, 410 W. 10th St., Suite 2000, Indianapolis, IN, United States

**Keywords:** Obesity prevention, Pediatrics, Qualitative research, Human-centered design

## Abstract

**Objective:**

How we communicate about obesity is critical as treatment paradigms shift upstream. We previously identified parental perceptions, concerns, beliefs, and communication preferences about early life obesity risk. We engaged parents of children 0 to 24 months of age and pediatricians from Indianapolis, Indiana, USA in the co-design of messages and tools that can be used to facilitate parent/provider conversations about early life obesity prevention.

**Methods:**

From April to June 2021, we conducted a series of co-design workshops with parents of children ages 0 to 24 months and pediatricians to identify their preferences for communicating obesity prevention in the setting of a pediatric well visit. Human-centered design techniques, including affinity diagraming and model building, were used to inform key elements of a communication model and communication strategy messages. These elements were combined and refined to create prototype tools that were subsequently refined using stakeholder feedback.

**Results:**

Parent participants included 11 mothers and 2 fathers: 8 white, 4 black, and 1 Asian; median age 33 years with 38% reporting annual household incomes less than $50,000. Pediatricians included 7 female and 6 male providers; 69% white. Through an iterative process of co-design, we created an exam room poster that addresses common misconceptions about infant feeding, sleep and exercise, and a behavior change plan to foster parent/provider collaboration focused on achieving children’s healthy weight.

**Conclusions:**

Our hands-on, collaborative approach may ultimately improve uptake, acceptability and usability of early life obesity interventions by ensuring that parents remain at the center of prevention efforts.

## 1 Introduction

Approximately 1 in 3 children in the United States has overweight or obesity, (Fryar et al., 2018) and rates of both worsened during the COVID-19 pandemic. (Lange et al., 2021) These children have increased risk of developing health problems including type 2 diabetes, hypertension, and cardiovascular disease. (Freedman et al., Sep 2001, Must and Strauss, Mar 1999, Dietz, Apr 2001, Taveras et al., 2008, Franks et al., 2010, Biro and Wien, May 2010, Sinha et al., 2002, Ogden et al., 2015) Early intervention is essential, as children with overweight and obesity are more likely to develop adult obesity and weight-related health conditions. (Taveras et al., 2011).

Pediatricians can help curb the obesity epidemic through screening, communication, and anticipatory guidance. The American Academy of Pediatrics recommends routine growth monitoring and screening for obesity-related comorbidities as part of routine primary care, (Daniels et al., 2015) but parents (Gillespie et al., 2015) and providers (Huang et al., 2011, Dinkel et al., 2017, Ray et al., Apr 2022) often avoid the subject. Along with time and resource constraints, providers report feeling uncomfortable raising the issue, uncertainty about how to communicate with families about weight, and concern about how families will respond. (Abdin et al., 2021, We Feel Like We Are in It Alone, xxxx, Walker et al., Sep 3 2007;, van Gerwen et al., Mar 2009, Perrin et al., 2005) This is further complicated with children less than 2 years of age, for whom pediatricians lack proper guidelines for obesity diagnosis and risk assessment. (Redsell et al., 2011) It is, perhaps, unsurprising that pediatricians desire better counseling tools to communicate weight problems to parents and help them achieve weight-related behavioral modifications. (Perrin et al., 2005).

This study is part of a multi-year project focused on identifying best practices for obesity prevention in early life. Previously, we conducted a needs assessment of parents of children ages 0 to 24 months to identify perceptions, concerns, beliefs, and communication preferences about early life obesity risk. (Cheng et al., 2022) Parents were open to obesity prevention intervention and guidance and believe pediatricians are the best, most trusted source of health information. However, few parents perceived excess weight in infancy as a health-related issue. We identified knowledge gaps and misperceptions parents had related to infant feeding and mobility; they also desired easy-to-understand, age-appropriate, and actionable information about recognizing fullness, active play, and healthy eating. Using these preliminary data as a guide, the objective of this study was to engage with parents of children 0 to 24 months of age, along with pediatric providers in the co-design of tools to facilitate pediatric provider-parent communication about obesity prevention.

## 2 Materials and methods

We collaborated with the Indiana Clinical and Translational Sciences Institute’s Patient Engagement Core, (What is Research Jam, xxxx) Research Jam (RJ), a multi-disciplinary team of trained researchers who apply human-centered design (HCD) techniques to health services research. (Sanematsu and Wiehe, 2014, Sanematsu and Wiehe, 2013, Sanematsu, January 2011 2011,) Study protocols were approved by our institution’s IRB.

### 2.1 Study participants

Our recruitment strategy is detailed elsewhere. (Cheng et al., 2022) Briefly, between July and December 2020 we recruited parents/caregivers of children ages 0 to 24 months via Amazon’s Mechanical Turk (mTurk), social media (e.g., Facebook and Reddit), and outreach to local organizations that serve children (e.g., IUPUI Center for Young Children, Indianapolis Tabernacle Presbyterian Church, the Women, Infants and Children (WIC) Program, the Indiana State Department of Health, and Indiana CTSI coalitions). Eligibility criteria included age at least 18 years, English proficiency, and US residence. Interested participants were directed to a RedCap system to provide electronic consent and demographic information, including a free text question to screen out bots. From this group, we selected a pool of participants to ensure representation across various ages of children and household incomes.

We recruited pediatricians by sharing study information with: (1) RJ’s email newsletter list which includes providers from previous studies; (2) RJ’s social media channels (e.g., Facebook, Instagram, and Twitter); (3) Connections IN Health, a local health coalition; (4) pediatric-focused Facebook and Reddit groups; and (5) ResearchMatch.org’s participant database. Eligible participants were recruited via the same process detailed above on a first come, first serve basis.

### 2.2 Data collection

Using HCD methodologies, we partnered with our parent and clinical stakeholders to co-create messages and tools to facilitate conversations about early life obesity prevention. HCD is increasingly being used within healthcare and is a participatory, iterative design process where stakeholders most closely affected by the problem are engaged in developing a solution. (Giacomin, 2014) Key practice components of HCD are building empathy, thinking by doing, making things visual, and fostering collaboration and empowerment amongst stakeholders. These tenets are critical to ensure that interventions resonate with intended participants, promote trust, and reduce stigma. (Domecq et al., 2014).

We designed multiple HCD activities with the ultimate goal of creating tools to help parents and pediatricians discuss obesity prevention during a pediatric well visit. Study activities took place online between April and June 2021 and focused on children under 24 months of age. The first activity instructed participants to write two scripts consisting of a pediatrician and a parent discussing a child’s weight. One script was a “drama script” in which “everything goes wrong” and the second was a “family show” script in which “everything works out at the end.” The goal was to identify key negative and positive elements of a communication strategy between parents and pediatricians about obesity risk. The second activity instructed participants to “create or find a visual aid” to help pediatricians discuss obesity prevention. Stakeholders were instructed to describe the aid and how it would be used to facilitate obesity-related conversations in this setting.

We then convened participants for a group Show-and-Tell Session to discuss a selection of the scripts and visual aids. The parent session took place via Zoom and lasted approximately 90 min. Due to scheduling challenges, pediatricians completed their Show and Tell asynchronously using FocusVision’s Revelation, (FocusVision, 2020) an online qualitative research platform we have used in prior work. (Cheng et al., 2022).

### 2.3 Data analysis

First, we established key aspects of a communication plan desired by the stakeholders. Data generated from the activities were downloaded into Miro, an online collaborative whiteboarding program. (Version 0.6.6., 2021) We separated discrete ideas, called “snippets”, onto digital sticky notes. Each snippet was reviewed by at least two researchers and disagreements were resolved by group discussion. The team then organized snippets based on how the content related to each other, creating an affinity diagram, a visual representation of data groupings and relatedness. (Kolko, 2011) Groupings were reviewed and revised by the team. Themes from parents and pediatricians were diagrammed separately and then merged into a single, color-coded affinity diagram for analysis.

Using the affinity diagram as a guide, we then established key messages about early life obesity prevention. We created a content map visualizing the ideal conversation flow that would occur between the pediatrician and parent. We identified specific communication guidelines derived from the Weaver communication model, (Fiske, 2010, Shannon, 1948) including: the sender (e.g., the most appropriate person to give the information), receiver (e.g., the intended recipient of the information), message (e.g., the content and tone of the information), media (e.g., the mechanism for delivering the information), and environment (e.g., the location where the recipient receives the information).

Finally, the team ideated to create rough prototype tools based on the above findings and recommendations and on our previous work. (Cheng et al., 2022) The goals of the prototype tools were to facilitate communication between parents and their pediatricians around early life obesity prevention and to address the educational gaps we identified, including: (1) common misconceptions parents have around healthy habits; (2) general recommendations for feeding, sleep, and exercise; and (3) how to interpret growth charts. Finally, we refined the prototypes after obtaining stakeholder feedback.

## 3 Results

Thirteen parents (11 mothers and 2 fathers) of children 0 to 24 months of age participated in at least one activity: median age 33 years; 8 white, 4 black, and 1 Asian. Nine were married, 3 were single/never married, and 1 was divorced; 38% reported annual household incomes below $50,000. Three parents did not complete the activities. Thirteen pediatricians completed all of the activities: median age 35 years, 69% white, and 54% female.

### 3.1 Ideal conversation flow

**Fig. 1:**
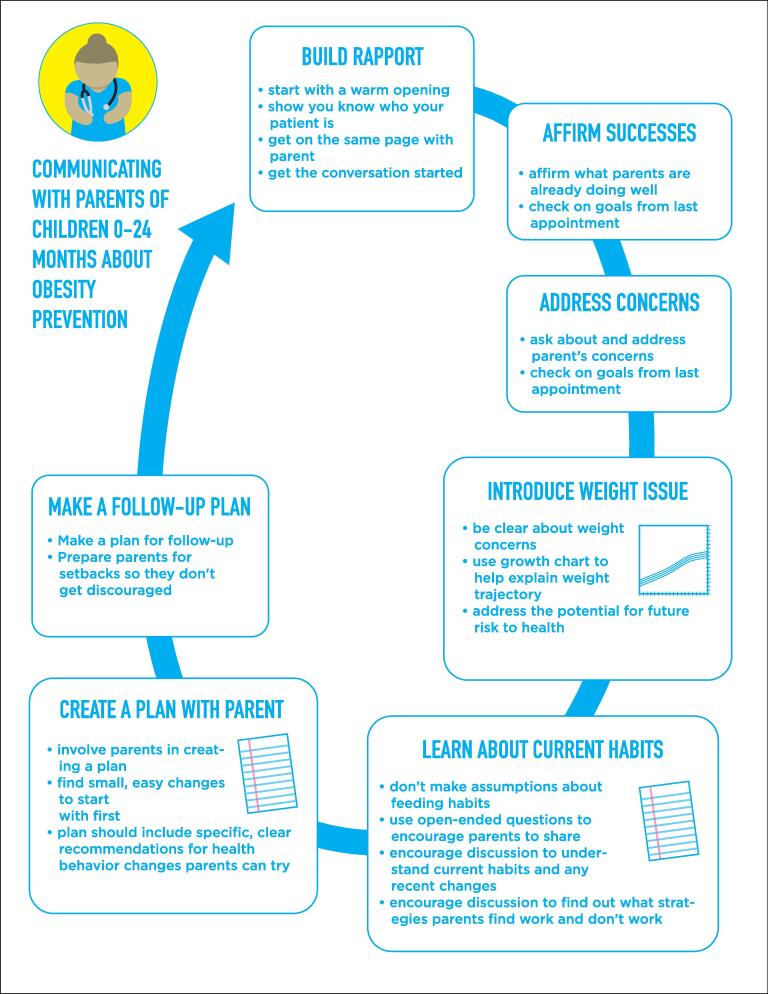
Final model showing the ideal steps of communicating obesity prevention information with parents of children age 0–24 months.

Fig. 1 shows key components of an ideal conversation about obesity prevention between parents and pediatricians. Stakeholders felt that a pediatric approach should:

**1. Build rapport**. Nearly all family show scripts began with rapport-building, wherein the pediatrician greeted the parent, asked how the parent and child were doing, and said something positive about the child.

“The pediatrician walks in with a big smile, and ask how the father and baby are doing? Also she looks right at the baby and talks in a baby friendly voice saying how cute the baby is. The father responds with everyone is doing good, and what they are working on at home with the baby. The father is smiling the whole time, and you can feel in the room how the doctor is showing attention, and how comfortable everyone is.” *- Excerpt from one of the parent family show scripts*

2. **Affirm success**. Stakeholders expressed that conversations should include statements about what parents are already doing well.

“First off, I want to say that I think you’re doing a wonderful job. Margaret looks happy and she’s meeting all her milestones.” *- Excerpt from one of the parent family show scripts*

“I liked how the doctor started off saying Margaret…met like all her milestones. She’s doing really good and everything. That probably, that would make the mom feel good about herself.”- *Parent during discussion of the family show scripts*

“(Healthy weight) is advice that isn’t always well received and often leaves the affirmation-needs unfulfilled.” - *Pediatrician during discussion of the family show scripts*

3. **Address general concerns first**. Stakeholders thought pediatricians should ask about the parent’s concerns before discussing the child’s weight.

“Address parental concerns 1st. Then move on to growth curve.” - *Pediatrician during discussion of the family show scripts*

**4. Introduce weight issues using growth charts and direct language**. Pediatricians advocated for using growth charts. Parents expressed a desire for pediatricians to be clear and up-front about the potential negative health outcomes that can result from obesity without being hyperbolic or over-emotional.

“A lot of times showing the parent the actual growth curve makes an impact, also plotting out the comparison of what age their weight would be average…sometimes it doesn’t seem like stating they are above the 99th makes an impression but realizing that the 5 year old has a BMI or weight of the average 10, 11, 12 year old really hits home.” - *Pediatrician during discussion of the family show scripts*

“I think maybe the doctor should have included the handout more about future health issues to be concerned about so that the parent has a better understanding of what we’re trying to prevent.” - *Parent during discussion of the family show scripts*

**5. Learn about current habits**. Many family show scripts included the pediatrician asking the parent about the child’s eating habits. During the Show-and-Tell, participants explained that this avoids making assumptions, helps identify recent changes that might affect the child’s weight, and helps ensure that recommendations are relevant and useful.

“It seems like your baby is growing pretty quickly. I want to make sure that we are doing all we can to start them out on a good healthy trajectory for their future and growth. What are their eating and play habits like?” *- Excerpt from one of the parent family show scripts*

“…asks an open-ended question that allows mom to give her thoughts/concerns rather than blaming or assuming the reason for the weight gain is over feeding.” - *Pediatrician during discussion of the family show scripts*

6. **Create a plan collaboratively with the parents**. Stakeholders identified collaboration as a good strategy versus pediatricians dictating what parents should do.

“It looks like he asked a lot of questions, but didn’t pause for any answers. And just said are you willing to hear what plans I have for him? Rather than maybe is there anything you think I could help you with to make, you know, remedy the situation?” - *Parent during discussion of the drama show scripts*

“I also thought it was good how the doctor asked if they could make a plan together.” - *Parent during discussion of the family show scripts*

“The pediatrician listened and needs to meet the parent where she’s at. She’d say, ‘It can be challenging to have a pickier eater. Luckily, he’s at the age where he can only choose to eat what you provide for him. I’ve never seen a child with access to food starve themselves.” *- Excerpt from one of the parent family show scripts*

**7. Make a follow-up plan**. Many stakeholders included a follow up plan in their activity scripts. These plans were simple and measurable, such as decreasing the number of ounces of breast milk or formula at each feeding.

“I like how in this iteration the doctor made his plan of action quantifiable, like measurable, [and] asked the mother to… record the child’s intake and the meals and stuff, so that it’s no longer subjective and it can be tracked, kind of as a way, had a plan of action with milestones, rather than just jostling with some advice.” *- Parent about one of the family show scripts*

### 3.2 Guidelines for communication

The team then identified ideal guidelines for discussing obesity prevention in the setting of the pediatric visit (Table 1). Having empathy and a non-judgmental approach were important to our parents. In terms of communicating messages, several parent scripts included pediatricians sharing personal anecdotes about their own children; in follow up discussions, parents said this made the pediatrician more relatable and credible. All stakeholders agreed that social stigma should not be used as a motivator for weight-related behavioral change and suggested making an explicit plan for follow up. Pediatricians advocated for using growth charts to guide weight-related conversations; parents desired pediatricians direct, unemotional language. Parents also wanted pediatricians to avoid comments about the weight of other family members, accusatory language, shaming, and using the word “diet.” They requested tools to: clarify healthy behaviors in children ages 0 to 24 months; give general guidelines for feeding, sleep, and exercise across this age range; assist parents in sharing current relevant behaviors, and create a written plan of action. Pediatrician stakeholders emphasized the need for these tools to fit into the context of a short well-child visit. Table 1 contains representative quotes from the participants regarding the function and context of these tools, derived from the script activity and the subsequent group Show and Tell.

**Table 1:** Communication guidelines to help pediatricians discuss obesity prevention with parents of children ages 0 to 24 months, findings from human-centered design activities with parents and pediatricians, Indianapolis IN, April-June 2021.

**DO….**	Excerpt from the Script Activity	Relevant Quote from the Show and Tell Activity
Share relevant personal parenting experiences	“If the pediatrician had a child of her own, she may **relate her own experiences to reinforce the point**, saying something like ‘My little Logan was the pickiest eater until he tried things at least five or ten times!’” [parent stakeholder]	“I always like it when the doctors feel like they can **relate to us and give us their own experience** on it, because it’s like, oh, okay, you’re not just telling me this because you’re a doctor. You’re also telling me because your mom as well, right? I know that would help me put it more in consideration, like yeah, okay, she’s a mom. She’s understands, it’s not that easy.” [parent stakeholder]
Show empathy	“The pediatrician **acknowledges** that kids this age can definitely be picky eaters and it can be a struggle to get them to eat healthy. She **reassures mom** that she knows that mom is doing the best that she can and that the child is otherwise healthy and developing well.” [pediatrician stakeholder] “Mom says: ‘He’s a picky eater. He fights me on eating healthier foods.’ The pediatrician responds by telling the parent ‘Most kids are!’ and ‘In fact, it usually takes several tries for a child to embrace the taste of a new type of food.’ The parent is **reassured and feels encouraged** that he or she may find success through persistence.” [parent stakeholder] “Dr.: Now I know you mentioned that your other daughter, Miranda, is starting pre-K and your husband just got a new job. With all that on your plate, I can imagine it can be a challenge with a little one like Margaret.” [parent stakeholder]	
Validate parents’ struggles and concerns	“The pediatrician **listened and needs to meet the parent where she’s at**. She’d say, ‘It can be challenging to have a pickier eater. Luckily he’s at the age where he can only choose to eat what you provide for him. I’ve never seen a child with access to food starve themselves.” [parent stakeholder]	
Prepare for set-backs, encourage small changes over perfection	“The pediatrician emphasizes that…she believes that the child may put up a bit of a fight about the decreased juice, but if mom **sticks with it**, the child will get used to it.” [pediatrician stakeholder] “Mom: ‘That sounds great. I still can’t dictate what they feed him at day care though.’ Pediatrician: ‘And **that’s okay**, we will work on a plan for home for now and then after we see how that is going we can see what we need to adjust at day care.’” [pediatrician stakeholder]	
Follow up	“We can **check in next visit to see how you’re doing**, whether this goal-setting has made things seem smoother and see how Margaret is doing.” [parent stakeholder] “It sounds like we have a bit of a **plan in place**. Why don’t you make an appointment in 1–2 months so we can follow-up on how everything is going?” [pediatrician stakeholder]	
**AVOID….**		
Social stigma	“Your baby’s weight is far too high for her age. What are you feeding her? Do you not worry about her health and if she stays this way **what will other kids and parents think!?**” [drama script, parent stakeholder] “**The other kids may make fun of him** if he is too heavy when he’s older, you don’t want that, do you?” [pediatrician stakeholder]	“Another good example of what not to do, is using the following as a reason to eat healthier ‘kids will make fun of him for his weight.’” [pediatrician stakeholder]
Judgement	“Wow look how chunky he has gotten, cute though! I’m glad you decided to come in today after missing two appointments. I have been trying to bring you in about Eric’s weight…My goodness **what have you been feeding him**?” [parent stakeholder] “She soon gets to feeding and nutrition and she just blurts out… ‘Your baby is really fat, by the way. **How did you let her get that way** already?! Don’t you know how to feed your kid?!’” [pediatrician stakeholder]	“It is human nature to be defensive when one is being attacked (whether actually or perceived as such). In this story, the pediatrician clearly sets a tone where the parent feels attacked and judged, **which makes progress in addressing the health issues at hand difficult**. It is important to take a non-judgmental viewpoint and attitude in approaching patients, especially so when dealing with topics that are known to be touchy/triggering.” [parent stakeholder]
Commenting on the weight of other family members	“**It seems you are overweight yourself** so I’m concerned that you’re pushing your unhealthy habits onto your child.” [parent stakeholder] “Mom: ‘I’m not worried about his weight now. I’ll worry if he’s overweight when he’s older.’ Pediatrician: ‘You know that might not be true if we **look at big brother**. You said yourself that you’re having trouble finding clothes that fit your older son. Weight problems start early.’” [pediatrician stakeholder]	
Hyperbole and emotional language	“You know you’re setting her up for **health complications and early death** right? Do you just not care about your child?” [parent stakeholder] “The weight is way too much. **He is going to [be] obese**. What do you feed him only junk food, probably no fruits or vegetables.” [pediatrician stakeholder] “[The pediatrician] soon gets to feeding and nutrition and she just blurts out… ‘**Your baby is really fat**, by the way. How did you let her get that way already?!’” [pediatrician stakeholder]	“…doctors should be professional. Do not involve too much emotional words.” [parent stakeholder]
Framing behavior change as a diet	“Mom: ‘Instead of focusing on weight, perhaps you can **provide strategies** to help my child maintain good health overall. If the weight continues to trend upward as he gets older, then we can focus on diet. Until then, he is too young.’” [parent stakeholder] “Mom: ‘Although I don’t think babies should have a restricted diet, I am open to **help with meal planning**, especially because babies and toddlers can be picky eaters.’” [parent stakeholder] “Mom: ‘Putting a baby on a diet is just wrong.’ Pediatrician: ‘I agree! **I definitely don’t want to put him on a diet**! But what I would like to do is to work with you to figure out how we can make his daily schedule and eating habits reflect the amount of food and activity that is healthy for him at this age.’” [pediatrician stakeholder]	
Accusations or shaming	“I have heard that excuse before and it is a lame one.” [pediatrician stakeholder] “Rather than presenting solutions as easy opportunities to improve the baby’s health, the pediatrician uses words and phrases that suggest the parent’s actions are the root cause on which to blame the baby’s obesity. Parent becomes defensive, perhaps argumentative, providing excuses and retort instead of addressing the child’s issue.” [parent stakeholder]	“Dr. Pepper starts with blaming attitude to the family from the start.” [pediatrician stakeholder] “I also liked that she didn’t take any kind of an accusatory tone. It was more of a let’s talk about what’s going on and how maybe we can modify it.”

**Fig. 2:**
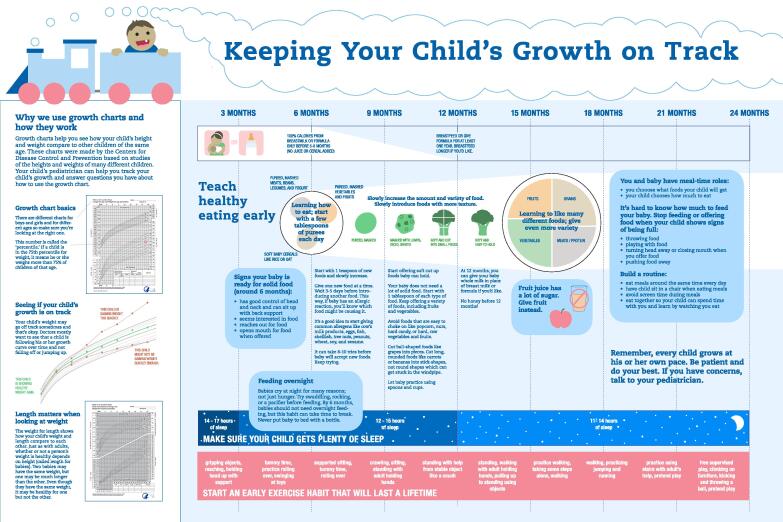

**Prototype tools**. The team developed two tools based on the above findings and recommendations: (1) an educational poster; and (2) a parent behavior change plan. The educational poster (Fig. 2) was designed to be viewed by parents in the exam room, prompting a discussion about obesity prevention with their pediatrician during the visit. The team designed the poster to increase general awareness of obesity prevention in early life, to provide health-related information, and to address the common misconceptions parents reported around healthy habits for young children, including general recommendations for feeding, sleep, and exercise. It also presents and explains growth charts to help parents interpret them.

**Fig. 3:**
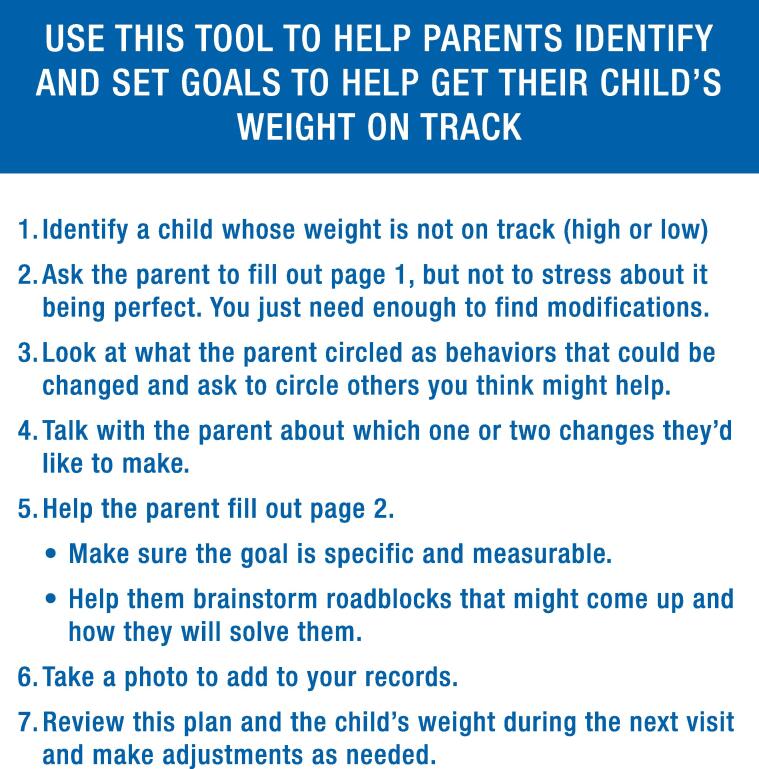

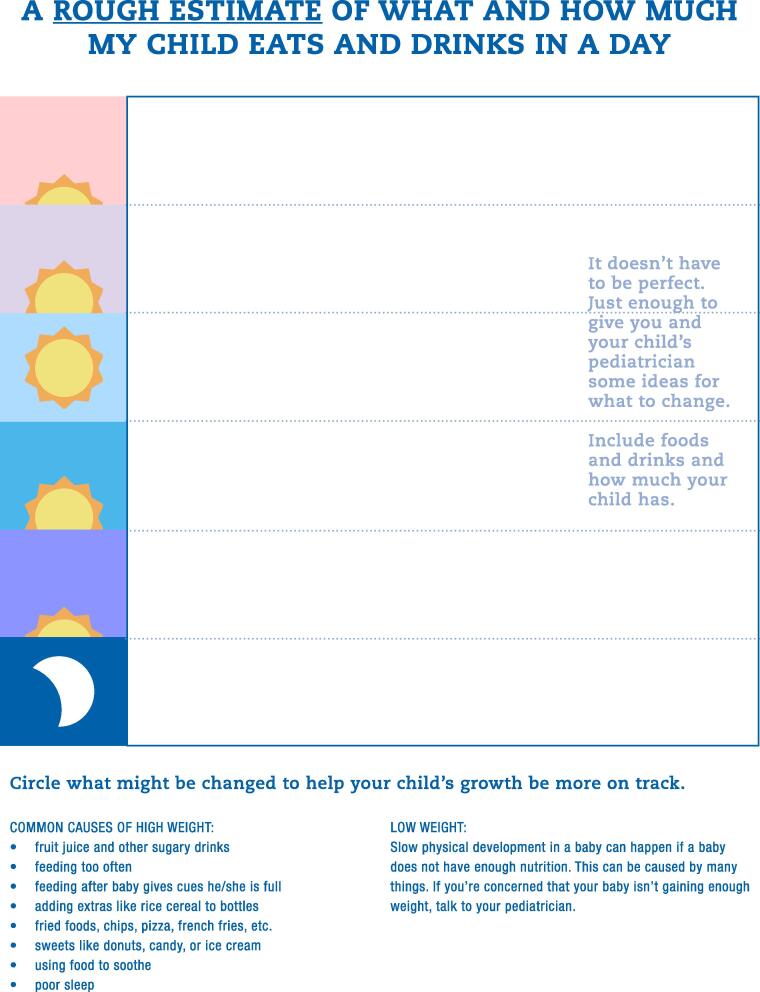

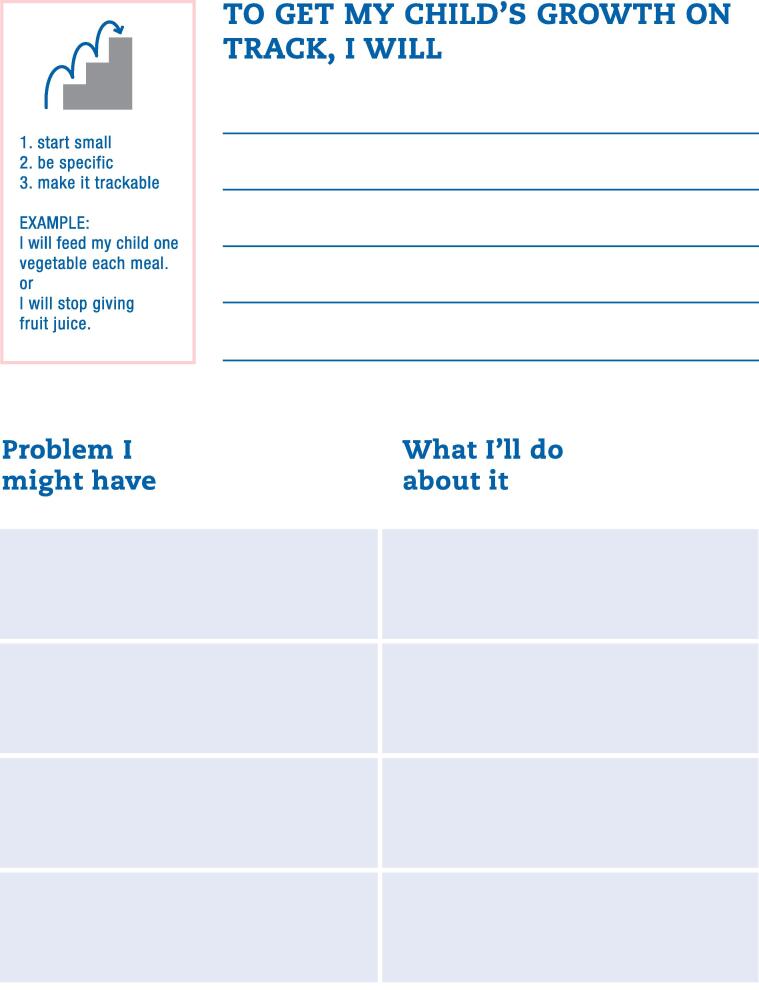

The second tool was a behavior change plan, which we developed because parents emphasized that achieving and maintaining their child’s healthy weight should be a “team effort” with their pediatrician (Fig. 3). The intent is for parents to complete the plan between visits, allowing pediatricians to identify areas for improvement, and prompting conversations with parents about goals for and progress towards optimal weight-promoting behavioral change.

## 4 Discussion

Using HCD, we engaged key stakeholders in the co-design of two tools to help facilitate parent/provider communication about obesity prevention in the pediatric office setting. The key functions of our tools, informed by stakeholders, were to address common misconceptions about health behaviors in children ages 0 to 24 months, to provide up-to-date and easy-to-understand guidelines about healthy-weight promoting behaviors, and to help build a parent/provider relationship focused on achieving and maintaining a child’s healthy weight.

In previous work,^23^ we learned that parents, although receptive to obesity prevention, were uncertain about early life obesity management, highlighting an opportunity for education. Encouragingly, parents were confident they could make good choices for their children and, when uncertain, relied on their child’s pediatrician for health advice. The regularity of well-child visits in the first years of life makes pediatricians ideally situated to monitor children’s weight over time and counsel families on obesity risk factors, although these topics are not always discussed. To facilitate discussions, we created a parent-informed conversation structure starting with building rapport (Fig. 1) and communication guidelines including avoiding judgmental, hyperbolic, and accusatory language (Table 1).

To guide weight-related discussions, pediatrician stakeholders preferred using growth charts, which are easily accessible and have been shown to assist care providers of older children objectively raise weight-related concerns. (Edvardsson et al., 2009, Toftemo et al., 2013) However, growth charts can be difficult for parents to interpret and may oversimplify or improperly label children if used without other counseling methods. (Hendrickson and Pitt, 2022) In qualitative work, providers desire more engaging and visually appealing educational resources about childhood feeding that incorporate up-to-date guidelines. (Heller et al., 2021) Growth charts are also limited because they do not focus on modifiable lifestyle behaviors or disease prevention, which our parents emphasized as having more relevance that weight alone. This, along with the educational gaps our parents identified, suggest that while growth charts should help anchor discussions, additional tools are needed.

Our tools address these needs. The first, an educational poster, provides a written explanation of growth charts to help parents interpret their child’s weight, as well as general, up-to-date guidelines that address common misconceptions parents of young children reported about feeding, sleep, and exercise. We selected an exam room poster because it is a low-cost way to educate parents about healthy growth and lifestyle behaviors that mirrors anticipatory guidance given by providers. A poster also utilizes downtime while parents wait to see their provider. Previous work shows that posters can be successful pediatric public health education tools, (Brown et al., Jul 2020, Herrera et al., 2013) and may help facilitate conversations about pediatric weight. (Brown et al., Jul 2020) We intend to examine whether our poster improves parent/provider communication about early life obesity prevention in future research.

Awareness of early childhood obesity prevention represents only one step in achieving healthy weight. Pediatricians can help ensure long-term, sustained change through ongoing communication, goal-setting, and consistent support and feedback. Therefore, the second tool we created serves as a written plan of action to assist parents and providers in achieving behavioral change. Our behavior change plan is intended to help parents and pediatricians collaboratively establish realistic, achievable weight-related goals in a non-stigmatizing way. We designed the behavior change plan to foster self-monitoring and developmentally-appropriate goal setting, two core strategies that can help families change their behaviors. (Faith et al., 2012) The tool avoids making assumptions about at-home behaviors and allows parents to lead the conversation by identifying opportunities for change before receiving suggestions from their pediatrician. We also developed this tool so that it can be combined with patient-centered counseling techniques successful in obesity interventions, such as motivational interviewing. (Barlow and Committee, 2007).

Ultimately, clinical settings must build a culture of obesity prevention rather than management, and change focus from weight to healthy lifestyle. Therefore, when using our tools, we urge providers to avoid stigmatizing language which may perpetuate weight bias, and to instead frame conversations in the context of other developmental milestones in alignment with our prior qualitative work. (Cheng et al., 2022) Framing weight-related conversations in this context and focusing on modifiable lifestyle behaviors rather than focusing on weight with parent preferences and appears most likely to influence parental receptiveness to engage in preventative interventions.

Our tools may also have utility for community-based programs that provide timely but sometimes conflicting (Heller et al., 2021) dietary guidance to families. Recent research notes that early education (e.g., childcare) and community-based (e.g., Head Start, WIC, home visiting) providers have limited access to evidence-based educational materials on childhood feeding, particularly for children ages 0 to 24 months, and desire engaging and visually appealing materials that address parental misconception of nutritional requirements for their children. (Heller et al., 2021) Our tools could address these gaps, providing consistent, evidence-based information about child feeding and other obesogenic behaviors not routinely addressed in these settings (e.g., sleep) thereby helping maximize the integration of obesity prevention efforts across multiple settings.

Limitations include a small sample size and low participation from minoritized populations and from lower income households. We did not translate our materials to other languages or adapt them to include culturally-appropriate foods and practices. Not all parents completed the activities. COVID-related stressors (e.g., food insecurity or feeding practices) (Adams et al., 2020) may have influenced parental responses, but we did not ask about this. Finally, we did not examine the feasibility of integrating these tools into routine practice. Our exam room poster is meant to be viewed while parents wait for their provider, but primary care visits are often rushed which may impact parents’ ability to ask follow up questions. Providers may have limited knowledge about local resources necessary for behavioral changes. We also did not examine other barriers, including work stress, burnout, and lack of reimbursement could prevent parent/provider engagement and the use of our tools. Next steps include evaluating the effectiveness, impact, and outcomes of using these tools to engage parents and physicians in early life obesity prevention.

There is a need for readily available, evidence-based, engaging patient education materials for provider interactions with parents of infants and toddlers to address obesity prevention, as well as guidance to help frame the conversation. To our knowledge, these are the first tools developed for early life obesity prevention using a HCD approach to engage key stakeholders in the development process. This hands on, collaborative approach may ultimately improve uptake, acceptability and usability of early life obesity interventions by ensuring that parents remain at the center of prevention efforts.

Disclosure of Funding

This work was supported in part by NIH Grants K01DK114383 and K24DK10598. Research Jam: Indiana Clinical and Translational Sciences Institute’s Patient Engagement Core (PEC) is supported by the National Center for Advancing Translational Sciences, Clinical and Translational Sciences Award [UL1TR002529].

## Declaration of Competing Interest

The authors declare that they have no known competing financial interests or personal relationships that could have appeared to influence the work reported in this paper.

## Data Availability

The data that has been used is confidential.
